# Transfer of human frozen-thawed embryos with further cleavage during culture increases pregnancy rates

**DOI:** 10.4103/0974-1208.69340

**Published:** 2010

**Authors:** Bharat V Joshi, Manish R Banker, Pravin M Patel, Preeti B Shah

**Affiliations:** Pulse Women’s Hospital, Ahmedabad, India

**Keywords:** Cleavage, embryo cryopreservation, pregnancy rate, thawing

## Abstract

**AIM:**

To compare the pregnancy rate following transfer of frozen-thawed embryos with or without overnight culture after thawing.

**SETTINGS AND DESIGN:**

This is a retrospective analysis of frozen-thawed embryo transfer (FET) cycles performed between January 2006 and December 2008.

**MATERIALS AND METHODS:**

Out of 518 thaw cycles, 504 resulted in embryo transfers (ETs). Of the total FET cycles, 415 were performed after an overnight culture of embryos (group A); and in 89 cycles, ET was performed within 2 hours of embryo thawing (group B).

**STATISTICAL ANALYSIS:**

The data were statistically analyzed using chi-square test.

**RESULTS:**

We observed that with FET, women ≤30 years of age had a significantly higher (*P*=0.003) pregnancy rate (PR=28.9%) as compared to women >30 years of age (17.5%). A significantly higher (P<0.001**) pregnancy rate was also observed in women receiving 3 frozen-thawed embryos (29%) as compared to those who received less than 3 embryos (10.7%). The difference in PR between group A (PR=24.3%) and group B (PR=20.3%) was not statistically significant. However, within group A, ET with cleaved embryos showed significantly (*P*≤0.01) higher pregnancy rate compared to the uncleaved embryos, depending on the number of cleaved embryos transferred.

**CONCLUSION:**

No significant difference was noticed between FETs made with transfer of embryos with overnight culture and those without culture. However, within the cultured group, transfer of embryos cleaved during overnight culture gave significantly higher PR than transfers without any cleavage.

## INTRODUCTION

The first report of human pregnancy through transfer of frozen and thawed embryos was published in 1983[1]; and a year after, the first birth through such transfer was reported.[2] In the last decade, there has been a series of developments in the field of assisted reproductive techniques (ARTs).[3] The pregnancy rates have reportedly been doubled between 1994 and 2003 despite a decrease in the mean number of embryos transferred.[4] These advances can easily be attributed to a better understanding of the embryo selection process, embryo transfer (ET) technique, ovarian stimulation and cryopreservation techniques.[3] As the advantages and the need of cryopreservation became obvious, cryopreservation has become an integral part of ART setup. These advantages include optimum use of embryos formed, with increased cumulative pregnancy rate; restriction of risks of multiple pregnancies and ovarian hyperstimulation; and cost effectiveness of the thaw cycles.[3,5] Since inception in 1996, embryo cryopreservation has been included in our ART program. One of the options to improve the results after frozenthawed embryo transfer (FET) is to consider when to transfer embryos after thawing i.e., either just after thawing or after culturing them *in vitro* to observe their growth and further development, as further cleavage of the blastomeres may predict their better implantation potential.

## MATERIALS AND METHODS

### Period and patients

The study involved a total of 518 thaw cycles performed over a period of 3 years, from January 2006 to December 2008, resulting in 504 FETs. The patients were between 22 and 48 years of age, with the mean age being 30.47 years. They were further divided into two groups: group A (*n*=415; mean age of patients, 31.6 years, ranging from 22 to 48 years), in whom embryos were transferred after an overnight culture; and group B (*n*=89; mean age of patients, 30.1 years, ranging from 22 to 42 years), in whom embryos were transferred within 2 hours of thawing. Patient selection criterion for overnight culture depended on whether they had more than 5 frozen embryos. Ovarian stimulation was achieved by using a gonadotrophin-releasing hormone (GnRH) analogue long protocol or antagonist protocol along with administration of gonadotrophin for 10 to 15 days. When a majority of the growing follicles were at least 18 mm in size, 10,000 IU of human chorionic gonadotrophin (hCG) was administered. The oocytes were collected 34 to 36 hours post–hCG administration. IVF / ICSI was carried out for insemination.

### Embryos

Morphological assessment of all embryos was carried out on day 2 post-insemination. The numbers of blastomeres were counted, and degree of cytoplasmic fragmentation was analyzed. The embryos were divided into three grades based on the extent of cytoplasmic fragmentation. On day 2 post-insemination, not more than three best embryos were transferred in the egg-retrieval cycle. All the surplus grade 1 and grade 2 embryos with ≤30% cytoplasmic fragmentation were used for cryopreservation.

### Cryopreservation

Embryos were first equilibrated using 1.5 mol/L of 1,2-propanediol for 10 minutes at room temperature. Subsequently, they were transferred to a solution of 1.5 mol/L of 1,2-propendiol and 0.1 mol/L of sucrose in phosphate buffer saline (embryo-freezing media, Vitrolife, Sweden). Immediately after transfer to freezing solution, embryos were loaded into plastic straws, and gradual cooling was carried out at the rate of −2°C/min to −8°C/ min in a programmable freezer (Cryologic, Australia). After holding for 5 minutes at −8°C, the embryos were manually seeded and subjected to further cooling to −35°C at the rate of 0.3°C/min. After a free fall to −120°C, the straws were plunged and stored in liquid nitrogen for cryopreservation.

### Embryo thawing and transfer

Embryos were thawed rapidly. Straws were exposed to air (room temperature) for 30 seconds and immersed in water at 30°C for 30 seconds. Cryoprotectants were removed in steps using embryo-thawing media (Vitrolife). Embryos were transferred to culture media before being assessed for the number of surviving blastomeres. The evaluation criteria for morphological survival of these frozen-thawed embryos were set as follows: ≥50% of the initial number of blastomeres intact and no signs of damage to the zona pellucida. All the embryos which fulfilled the above criteria were transferred either within 2 hours of thawing (on day 2 post-insemination, group B) or after culturing these overnight (on day 3 post-insemination, group A). In the latter group, embryos were checked for further cleavage.

### Patient preparation and pregnancy outcome

Endometrium of patients was prepared with Estradiol valerate (Progynova^®^ Scherring, Sikkim, India) 4 mg/d from the second day of the menstrual cycle for 4 days; and then, 6 mg/d. It was increased to a maximum of 8 mg/d if required till the endometrial thickness exceeded 8 mm. Micronized progesterone (Puregest^®^, Sun Pharmaceuticals, Halol, India) 200 mg/d thrice a day was added and ET carried out 2 days later. Pregnancy was confirmed by detecting blood levels of Beta-Human Chorionic Gonadotrophin (βhCG) on the 15^th^ day after ET. A βhCG level ≥100 mIU/mL was considered positive. In case βhCG value was >10 or <100 mIU/mL, the detection test for βhCG was repeated after 48 hours. The presence of a gestational sac was confirmed 1 week after positive βhCG by transvaginal ultrasound examination.

### Statistical analysis

Four factors were analyzed using chi-square analysis for their influence on the overall pregnancy rate, namely, patient age, number of embryos transferred, culture duration, number of cleaved embryos at transfer.

## RESULTS

### Embryo survival

In this retrospective study, out of 518 thaw cycles, 504 (97%) resulted in ET. A total of 2,241 embryos were thawed in these cycles, of which 1,343 survived the procedure and were found suitable for transfer, with a survival rate of 59.9%. Of the 1,113 embryos cultured overnight, in 530 (47.6%) the resumption of mitosis through further cleavage was observed.

### Pregnancy rate according to age of patients

As no significant difference was observed between the two groups (A and B), all the patients were included in analyzing other factors. Patients aged ≤30 years had a significantly higher (*P*=0.003) pregnancy rate (28.9%) [Table 1] as compared to the patients >30 years of age (17.5%).

**Table 1:** Pregnancy rates according to patients’ age

Patient’s age (Years)	Number of ETs	Number of pregnancy	PR (%)	*P* value	
≤30	270	78	28.9	.003	
>30	234	41	17.5		

### Pregnancy rate according to number of embryos

Patients that received <3 embryos (10.7%) had a significantly lower rate of pregnancy as compared to patients that received 3 embryos (29%) [Table 2].

**Table 2:** Pregnancy rates according to number of embryos transferred

Number of embryos at ET	Number of ETs	Number of pregnancy	PR (%)	*P* value
Up to 2	149	16	10.7	<.001**
3	355	103	29.0	

### Pregnancy rate according to time of ET

Although a higher pregnancy rate was observed in group A (24.3%) compared to group B (20.3%), yet it was statistically not significant [Table 3].

**Table 3:** Pregnancy rates according to time of ET

Group	Mean age (Years)	Number of pregnancy/ ETs	PR (%)
A (overnight culture)	31.6	101/415	24.3
B (2 hours culture)	30.1	18/ 89	20.3

### Pregnancy rate according to cleavage

Further analysis of ETs with cultured embryos revealed that highly significant PRs were observed when ≥2 cleaved embryos were transferred (42.2%) than when 1 cleaved embryo (16.9%, *P*=0.0002) or embryos without cleavage were transferred (3.2%, *P*≤.0001) [Table 4].

**Table 4:** Pregnancy rates according to number of cleaved embryos

Number of cleaved embryos transferred	PR (%)	*P* value
0	3.22	<.0001[1,3]
1	16.9	.0002[2,3]
≥2	42.2	

## DISCUSSION

The aim of this retrospective analysis was to evaluate the influence of the thaw transfers made just after thawing of the embryos or after an overnight culture on the subsequent pregnancy rate.

An overall pregnancy rate of 23.6% with transfer of frozen-thawed embryos in this analysis is equivalent to pregnancy rates reported earlier.[6–8] An acceptable embryo survival rate of around 60% observed by us coincides with that observed by others.[7,9] Our finding of 47.6% cleavage rate of the thawed embryos during post-thaw culture period is higher than the 27%[7] and 33%[10] reported earlier.

Age of the patient plays a major role in determining pregnancy outcome, which is also evident in our results and is an extension to the earlier published reports.[6] Similarly, our results established that the number of embryos transferred per cycle also plays a significant role, as observed in earlier studies.[6,8]

Comparison of the results with regard to the duration of culture showed no significant difference between group A (embryos cultured overnight after thawing) and group B (embryos transferred within 2 hours of thawing). However, when the results in group A were further analyzed according to the number of cleaved embryos used for transfer, a significantly higher pregnancy rate was observed when 2 or more cleaved embryos were transferred compared to ETs with only 1 cleaved embryo or ETs with uncleaved embryos. These results, although higher, are in agreement with the results previously reported.[7]

## CONCLUSION

In order to further improve the pregnancy rates after cryopreservation, we need to focus on selection criteria for the thawed embryos. In assisted reproduction, selection of the right embryos for transfer remains the key factor for getting best outcomes. So far, more importance has been given to the selection of the embryos prior to cryopreservation[11–14]; however, post-thaw selection of embryos also plays an equally important role. The results indicate that pregnancy rates after cryopreservation may be significantly improved by selecting the right embryos for transfer. In our study, it is clear that cleavage capacity is a good indicator of embryo viability in terms of pregnancy outcome.
